# Anticholinergic Drugs and Oral Health-related Quality of Life in Patients with Schizophrenia: A Pilot Study

**DOI:** 10.1515/tnsci-2020-0003

**Published:** 2020-02-11

**Authors:** Justine Chapuis, Francesca Siu-Paredes, Claire Pavageau, Gilles Amador, Nathalie Rude, Frédéric Denis

**Affiliations:** 1 University Hospital of Tours ,Odontology Department, 37170 Chambray-lès-Tours, France; 2 EA 481 Integrative Neurosciences and Clinical. University Hospital of Besançon, 25000 Besançon, France; 3 Université Champagne Ardenne. Faculté d’odontologie de Reims, 51100 Reims, France; 4 University Hospital of Tours. Service d’odontologie du CHU de Tours, 37170 Chambray-lès-Tours, France; 5 Université de Nantes, Faculté d'Odontologie de Nantes, 44000 Nantes, France; 6 EA 75-05 Education, Ethique, Santé. Université de Tours, Faculté de Médecine, 37032 Tours, France

**Keywords:** oral disease, oral health, schizophrenia, anticholinergic drugs, oral health related quality of life

## Abstract

**Objective:**

The aim of this study was to explore, in a sample population of people with schizophrenia (PWS), the role of the anticholinergic burden on the perception of oral health-related quality of life (OHrQoL) in France.

**Methods:**

A pilot study was performed between March 2014 and January 2016. PWS were recruited from a population in Côte d’Or department in France. Dental status was investigated using the Decayed, Missing, or Filled Teeth (DMFT) index, the Xerostomia Index (XI), and the Global Oral Health Assessment Index (GOHAI) for OHrQoL. The anticholinergic impregnation score was recorded using the anticholinergic impregnation scale (AIS).

**Results:**

A sample of 62 people was selected. The DMFT score was 16.5± 8.7, the XI score was 22.9±7.8, the GOHAI score was 43.0±8.8, and the AIS score was 3.1±2.8. In total, 169 drugs were prescribed to the people of our sample, and 114 different anticholinergic drugs were observed. The most frequently used anticholinergic drugs (51.40%), in the study had a low antimuscarinic potency (1 point according to AIS scale). The multiple linear regression model showed that the OHrQoL scores were significantly lower when the DMFT scores, XI score, and anticholinergic scores were high.

**Conclusions:**

This pilot study highlighted the potential role of the anticholinergic burden on the OHrQoL of PWS. A study with a validated specific scale for the OHrQoL and a standard anticholinergic burden scale should be conducted to clarify the role of anticholinergic drugs on the OHrQoL for PWS.

## 1 Introduction

Anticholinergic drugs are prescribed for a wide range of conditions such as overactive bladder, chronic obstructive pulmonary disease, nausea and vomiting, depression and psychosis [1]. Anticholinergics are drugs that block the action of acetylcholine and can cause a variety of peripheral side effects, such as dry mouth caused by a reduction in the flow of saliva, and central side effects, such as cognitive impairment or worsening of tardive dyskinesia [2]. Harmful cumulative effects from anticholinergic drugs might have already impacted people with one clinical condition [3]. Furthermore, anticholinergic adverse effects increase with increasing the dose, and multiple low-level anticholinergic drugs can add up to the same anticholinergic burden (or more) than a single high-level anticholinergic drug [4, 5]. This potential for harm increases with frailty and age [6], and the anticholinergic drug use is closely related to serious negative outcomes for people using these drugs [7]. As far as we know, various anticholinergic burdens or risk scales have been devised, but no single standard anticholinergic burden scale stands out [8, 9, 10, 11, 12, 13, 14, 15].

Antipsychotic drugs represent the mainstay for the pharmacologic treatment of people suffering from schizophrenia. All antipsychotics are dopamine D2 receptor antagonists and induce blockage of dopaminergic neurotransmission in subcortical areas either acutely or during chronic treatment and they can also block other receptors such as histamine-1, muscarinic-1 and alpha-1 [16, 17]. The extrapyramidal side effect of treatment is more pronounced with the first-generation antipsychotics (FGA), such as haloperidol or chlorpromazine, than with the second-generation antipsychotics (SGA), such as aripiprazole or olanzapine [16, 17]. However, the blockage of D2 receptors is also a way to treat positive symptoms of schizophrenia.

Schizophrenia is a severe mental disorder characterized by delusions, hallucinations, and other cognitive difficulties [18]. Furthermore, people with schizophrenia (PWS) often neglect their self-care and suffer often from conditions such as dry mouth. Dry mouth can be attributed to the medications used to manage schizophrenia and to somatic disorders like type 2 diabetes [19, 20]. Smoking can also be an attributing factor for dry mouth. Furthermore, psychiatric patients are known to smoke more often than general population [21].

People in the general population who suffer from dry mouth clearly express difficulty speaking, eating and swallowing. Some patients complain of halitosis, chronic burning sensation, altered taste and intolerance to spicy food [22]. In general terms, the oral health-related quality of life (OHrQoL) of patients with dry mouth could be affected [23, 24]. However, PWS often fail to recognize their health needs and delay seeking advice or treatment [25]. We frequently observe wider variations between a patient’s perception and an objective evaluation of the patient’s dental status among PWS [26]. In this situation, it is difficult for caregivers to fully manage oral health needs.

In a previous study, we found that the Global Oral Health Assessment Index (GOHAI) exhibited excellent psychometric characteristics among PWS and could be used to assess the OHrQoL of these patients [27].

In this case, the role of dry mouth on the OHrQoL and how PWS perceive the side effects of these drugs need to be clarified before preventive recommendations and support for managing the oral side effects of psychotropics could be initiated.

The aim of this study was to explore, in a sample population of PWS in France, the role of anticholinergic burden drugs on the perception of the OHrQoL of these patients.

## 2 Material and methods

### 2.1 Design

A pilot study to explore the OHrQoL of PWS was performed between March 2014 and January 2016 from a sample population of PWS. PWS were recruited from a population of 1,868 PWS in Côte d’Or recorded in the French medico-administrative database, PMSI (Programme de Médicalisation des Système d’Information) of the three hospitals of Côte d’Or using a random stratified method. The sampling strategy of this study was described in a previous study by our team [28].

### 2.2 Population

Patients were included if they were over 18-years-of-age with a diagnosis of schizophrenia (Diagnosis: F20-F29 PMSI) according to the International Classification of Diseases 10th Revision [29] and were psychiatrically stable. People who could not understand or had a poor understanding of French were excluded.

### 2.3 Sample size estimation

For a pilot study, Browne et al. [30] suggested at least 30 subjects to estimate a parameter, whereas Teare et al. [31] suggested a sample size of approximately 70 in order to reduce the imprecision around the estimate of the standard deviation.

**Ethical approval:** The research related to human use has been complied with all the relevant national regulations, institutional policies and in accordance the tenets of the Helsinki Declaration, and has been approved by the Committee for the Protection of Persons (CPP) I of Eastern France (registration number: 2014-A00358-39). The study was registered on www.ClinicalTrials.gov under number NCT02167724.

**Informed consent:** Informed consent has been obtained from all individuals (or from their legal guardians for persons under guardianship) included in this study.

### 2.4 Outcomes

Age and prescribed drugs at the time of the examination were extracted from institutional medical records.

- Dental condition

Caries experience was assessed using the dentinal (D3) level and the Decayed, Missing, or Filled Teeth (DMFT) index based on 32 teeth and was calculated using the WHO (World Health Organization) criteria. The DMFT index is the sum of decayed teeth + missing teeth + filled teeth in the permanent dentition [32].

A single investigator, a dentist, interviewed and clinically examined all the participants using portable dental equipment at the hospital nearest to the subject’s place of residence. Ratings were calibrated in comparison with repeated examinations of a separate pilot sample. Kappa scores of 0.9 for inter-rater agreement were achieved.

- Oral Health Assessment

The Global Oral Health Assessment Index (GOHAI)

The GOHAI is a self-assessment oral health index. A French version has been validated with PWS [18]. The questionnaire consists of 12 questions that are phrased positively or negatively. The answer modalities are based on a Likert scale with scores ranging from 1 to 5. The GOHAI score is the sum of the answers to the 12 questions. According to Atchison and Dolan [33], a score of 57 to 60 is regarded as high and corresponds to a satisfactory oral health, quality of life. A score from 51 to 56 is regarded as average, and a score of 50 or less is regarded as a low score, reflecting a poor oral health quality of life.

- The Xerostomia Inventory (XI)

The Xerostomia Inventory (XI) is a self-assessment questionnaire. The XI is an 11-item summated rating scale that combines the responses to 11 individual items into a single continuous scale score, which represents the severity of chronic xerostomia; higher scores represent more severe symptoms. Respondents are asked to choose one of five responses (‘Never’, scoring 1; ‘Hardly ever’, 2; ‘Occasionally’, 3; ‘Fairly often’, 4; and ‘Very often’, 5). Each individual’s responses are scored and summed to give a single XI score [34].

- The anticholinergic impregnation scale (AIS)

The anticholinergic impregnation scale (AIS) is a specific anticholinergic burden scale for drugs used in France [35]. A score was assigned, ranging from 1 to 3 (1: low anticholinergic potential; 2: moderate anticholinergic potential and 3: high anticholinergic potential) to a list of 128 drugs with a consensus approach obtained via literature data and expert opinions. Anticholinergic impregnation score is the sum of both regularly used and when-needed drugs.

### 2.5 Data analysis

Qualitative variables were expressed as percentages, and quantitative variables were expressed as the means and standard deviations.

Wilcoxon nonparametric tests were used for the comparison of means.

A multiple linear regression model was used to explore the relationship between the GOHAI scores and the XI scores, the DMFT scores and the AIS scores.

The statistical significance level was set to p<0.05.

All statistical analyses were performed using SAS statistical software (version 9.1, SAS Institute, Cary, N.C.).

## 3 Results

### 3.1 Descriptive data

A sample of 62 people was selected. The population characteristics are reported in Table 1.

**Table 1:** Characteristics of sample studied (n=62)

	n (%)	m (sd)
**Demographic**		
Age (years)		43.7 (11.5)
Female	19 (30.6)	
Male	43 (69.4)	
**Treatment**		
Antipsychotic drugs		1.7 (0.9)
Anticholinergic drugs		2.9 (1.7)
AIS score		3.1 (2.8)
**Dental condition**		
GOHAI score		43.0 (8.8)
XI Score		22.9 (7.8)
DMFT		16.5 (8.7)
*Decayed*		*2.1 (3.2)*
*Missing*		*7.6 (9.5)*
*Filled*		*6.7 (5.7)*

Sd : standard deviation ; GOHAI : Global Oral Health Assessment Index; XI: Xerostomia Inventory; DMFT: decayed-mising-filled teeth.

In total, 169 drugs were prescribed to the patients of our sample, and 114 drugs were identified with a potential anticholinergic side effect. The most frequently used anticholinergic drugs (51.4%) used in the study had a low antimuscarinic potency (1 point according to AIS scale), 31.4% of the prescribed drugs had an AIS=3, and 17.1% had an AIS score of 2. The most prescribed anticholinergics drugs and their AIS scores are shown in Table 2.

**Table 2:** Anticholinergics drugs prescribed and AIS scores (n=114/169)

INN	AIS score	n (%)	Therapeutic class	ATC-codes
Halopéridol	1	17 (10. 0)	Antipsychotic	N05AD01
Tropatépine	3	17 (10. 0)	antiparkinsonian	N04AA12
Cyamémazine	3	16 (9.5)	Antipsychotic	N05AA06
Loxapine	2	14 (8.3)	Antipsychotic	N05AH01
Olanzapine	2	12 (7.1)	Antipsychotic	N05AH03
Rispéridone	1	10 (5.9)	Antipsychotic	N05AX08
Clozapine	3	8 (4.7)	Antipsychotic	N05AH02
Levomépromazine	2	8 (4.7)	Antipsychotic	N05AA02
Lorazépam	1	6 (3.6)	Anxiolytic	N05BA06
Paroxétine	2	6 (3.6)	Antidepressant	N06AB05

INN: International Nonproprietary Names; AIS: Anticholinergic Impregnation Scale; ATC-codes: Anatomical Therapeutic Chemical Classification System

### 3.2 Data analysis

The results of variables comparison two-by-two are shown in Table 3. There is a significant negative correlation (p <0.001) between the GOHAI score and the XI score and between the GOHAI and the DMFT score (p <0.001).

**Table 3:** Linear regression of variables two by two

	r	p
GOHAI vs DMFT	0,54	< 0,001
GOHAI vs XI	0.74	< 0,001
GOHAI vs AIS	0.0009	
AIS vs XI	0.12	

GOHAI: Global Oral Health Assessment Index; XI: Xerostomia Inventory; DMFT: decayed-missing-filled teeth; AIS: Anticholinergic Impregnation Scale; P values

The multiple linear regression model of factors associated with the GOHAI score are presented in Table 4. The GOHAI score was significantly lower when the DMFT score (p = 0.01), the CIA score (p = 0.05), the XI score (p = 2x10-16) and the age (p = 0.03) were higher.

**Table 4:** Factors associated with GOHAI score

GOHAI scores	Age m(sd) p = 0,03	AIS scores m(sd) p = 0,05	DMFT m(sd) p = 0,01	Xi Scores m(sd) p = 2x10-16
12≤GOHAI<35	45,2 (9,6)	6,0 (3,1)	22,8 (8,1)	32,0 (5,8)
35≤GOHAI<45	46,7 (10,7)	5,1 (3,8)	21,1 (6,8)	26,1 (7,1)
45<GOHAI<50	39,7 (9,4)	4,5 (3,4)	10,7 (6,1)	19,2 (5,9)
50≤GOHAI≤60	44,1 (14,8)	6,1 (3,3)	13,5 (8,4)	17,1 (4,0)

Sd : standard deviation; GOHAI : Geriatric Oral Health Assessment Index ; AIS: Anticholinergic Impregnation Scale ; DMFT: Decayed-Mising-Filled teeth; XI : Xerostomia Inventory; P-values; OHrQoL: Oral Health-related Quality of Life

## 4 Discussion

In our study, we highlighted that anticholinergic drugs take part in the OHrQOl of PWS like the dental condition, age and the xerostomia perception. But we can’t precise the impact of anticholinergic drugs in OHrQoL of PWS. The impact of anticholinergic drugs on oral health-related quality of life is a well-known matter, particularly among older people, but the impact of these drugs on the OHrQoL and dental diseases is often overlooked among people who suffer from severe mental illness, such as schizophrenia. We observed that the PWS sex ratio of our sample was in accord with the incidence of schizophrenia, which is significantly higher in males than in females [36]. The DMFT score was higher than in countries with the same income levels as France. For example, in Spain with persons suffering from schizophrenia, DMFT was 13.5±7.2 with a mean age of 40±11.2 vs. 16.5±8.7 [37] and was higher than the general population in France [38]. Many drugs prescribed for PWS show anticholinergic activity, and relationships seem to be emerging between a poor OHrQoL, the DMFT score, the XI score and anticholinergic drugs, but it was difficult to distinguish them from each other.

The OHrQol that includes dry mouth as evaluated by the XI scale is a complex and subjective multidimensional concept. The OHrQoL concept integrates several dimensions, such as functional limitation, physical pain, psychological discomfort, and physical, psychological and social disability or handicap related to the presence of oral disorders. But, side effects of antipsychotics are not directly evaluated by the GOHAI [27]. In other words, oral health is defined differently by each person according to his/her understanding of what a healthy mouth is, the type of symptoms previously experienced, cultural values, past experience with the health care system (regular visit at the dentist or not), general health, psychosocial wellbeing, impact of severe mental illness, age or gender [39]. Furthermore, schizophrenia leads to disturbances in the progression of thought, errors in contextual analysis and errors of logic. Often PWS do not recognize their health needs and delay seeking advice or treatment [40]. The neglect of self-care by PWS appears to be influenced predominantly by negative symptoms of schizophrenia, such as lack of initiation, a lack of concern for personal health, social withdrawal and a lack of motivation [41]. Studying the social construction of the illness, the characteristics related to quality of life and the description of the determinants of health inequalities in individuals suffering from this disease is important [42]. Nevertheless, qualitative research on this topic is scarce. Specific tools to assess the OHrQoL for PWS, including the side effects of antipsychotics, should be useful for a better understanding and management the oral side effects of medications used to treat mental illness [43].

Evidence is accumulating in relation to a range of adverse outcomes associated with using multiple drugs with anticholinergic properties for PWS in term of their OHrQol [2, 3, 4, 5, 6, 7, 8, 9, 10, 11, 12, 13, 14, 15, 16, 19, 20, 21, 22, 23]. To the best of our knowledge, no other studies have been conducted using a method to quantify the cumulative effect of taking multiple drugs with anticholinergic properties that addressed the perception of dry mouth or side effects of these drugs in the OHrQol of PWS.

In the literature, a wide range of anticholinergic burden scales have been devised for the older population to aid in medication reviews so that certain drugs can either be stopped, or the medication regimen altered [4447]. In this study, we chose the AIS because this is a scale that considers drugs used in France and is based on an assessment of the drugs used together with the peripheral anticholinergic side effects. However, there is no single standard anticholinergic burden scale to aid in conducting medication reviews. Furthermore, most scales are constructed using expert opinion panels and do not take into account the perception of the OHrQoL of concerned people. However, the limitations include also differences in exposure to medicines, dosing, route of administration and false positives [44, 45, 46, 47].

In mental health, prescribers can use both FGA and SGA compounds at doses that result in levels between the domains on the dose–response curves for beneficial and anticholinergic effects. This dose-dependent therapeutic domain differs not only between drugs but also between patients. The effect of a certain dose of a certain drug on an individual is difficult to predict, as it depends on several and very different parameters, such as the magnitude of the compensatory response, the level of tolerance, the subject’s state of health and the history of the drug administration [48].

## 5 Limitations

First, properties of anticholinergic drugs may cause peripheral or central side effects that hard to distinguish from illness-related symptoms [3]. The GOHAI identify more oral functional than psychosocial impact and these measures are mainly assessing the consequences of dental conditions rather than the PWS’s perception and understanding of the chronicity of their underlying oral disease [27]. In other words, it is difficult to determine whether it is the condition (schizophrenia) or the medication that affects the oral health-related quality of life.

Second, the AIS scale was specifically designed to evaluate the anticholinergic burden in a French setting. Typically, French drugs, such as cyamemazine are evaluated with AIS scale [35]. But for example, this molecule is not marketed in the United States. There is a lot of scales to measure anticholinergic burden but not all the lists include the same drugs, and the points given for certain drugs differ among them [8, 9, 10, 11, 12, 13, 14, 15]. Furthermore, when-needed drugs are problematic when added into these scales due to their possible irregular usage and effects. Thus, they might cause falsely high anticholinergic burden, if their use is not regular. Finally, they are no agreement between scales to measure the anticholinergic burden. It is likely that our results should be different if we have used another scale to measure the anticholinergic burden.

In this study, the hypersalivation paradoxical side effect of clozapine [50] is not specifically highlighted by the AIS scale and no item of the GOHAI investigate discomfort induced by hypersalivation and it’s impact in OHrQoL.

Finally, in view of our small sample, we could not discriminate any different results between PWS suffer from positive or negative symptoms.

## 6 Conclusion

This pilot study highlighted the potential role of anticholinergic drugs on the OHrQoL of PWS. However, in order to help clinicians take into account the side effect profiles when choosing an agent, a study with a validated specific OHrQoL scale and a standard anticholinergic burden scale should be conducted to clarify the impact of anticholinergic drugs on the OHrQoL for PWS.

## List of Abbreviations

PWS: people with schizophrenia
OHrQoL: Oral Health related Quality of Life
DMFT: Decayed, Missing, or Filled Teeth
XI: Xerostomia Index
GOHAI: Global Oral Health Assessment Index
AIS: anticholinergic impregnation scale
FGA: first-generation antipsychotics
SGA: second-generation antipsychotics
